# miR-216a inhibits osteosarcoma cell proliferation, invasion and metastasis by targeting CDK14

**DOI:** 10.1038/cddis.2017.499

**Published:** 2017-10-12

**Authors:** Quanbo Ji, Xiaojie Xu, Ling Li, Stuart B Goodman, Wenzhi Bi, Meng Xu, Yameng Xu, Zhongyi Fan, William J Maloney, Qinong Ye, Yan Wang

**Affiliations:** 1 Department of Orthopaedics, General Hospital of Chinese People’s Liberation Army, Beijing, China; 2 Department of Orthopaedic Surgery, Stanford University, Stanford, CA, USA; 3 Department of Medical Molecular Biology, Beijing Institute of Biotechnology, Palo Alto, China; 4 Department of Traditional Chinese Medicine, Xinhua Hospital Affiliated to Shanghai Jiao Tong University School of Medicine, Shanghai, China; 5 Department of Oncology, General Hospital of Chinese People’s Liberation Army, Beijing, China

## Abstract

Osteosarcoma (OS) has emerged as the most common primary musculoskeletal malignant tumour affecting children and young adults. Cyclin-dependent kinases (CDKs) are closely associated with gene regulation in tumour biology. Accumulating evidence indicates that the aberrant function of CDK14 is involved in a broad spectrum of diseases and is associated with clinical outcomes. MicroRNAs (miRNAs) are crucial epigenetic regulators in the development of OS. However, the essential role of CDK14 and the molecular mechanisms by which miRNAs regulate CDK14 in the oncogenesis and progression of OS have not been fully elucidated. Here we found that CDK14 expression was closely associated with poor prognosis and overall survival of OS patients. Using dual-luciferase reporter assays, we also found that miR-216a inhibits CDK14 expression by binding to the 3′-untranslated region of CDK14. Overexpression of miR-216a significantly suppressed cell proliferation, migration and invasion *in vivo* and *in vitro* by inhibiting CDK14 production. Overexpression of CDK14 in the miR-216a-transfected OS cells effectively rescued the suppression of cell proliferation, migration and invasion caused by miR-216a. In addition, Kaplan–Meier analysis indicated that miR-216a expression predicted favourable clinical outcomes for OS patients. Moreover, miR-216a expression was downregulated in OS patients and was negatively associated with CDK14 expression. Overall, these data highlight the role of the miR-216a/CDK14 axis as a novel pleiotropic modulator and demonstrate the associated molecular mechanisms, thus suggesting the intriguing possibility that miR-216a activation and CDK14 inhibition may be novel and attractive therapeutic strategies for treating OS patients.

Osteosarcoma (OSA), the most common primary bone malignancy, is characterized by a wide spectrum of complicated pathologies and frequent distal metastasis, and causes death in adolescents and young adults worldwide.^1, 2, 3^ Patients with localized OS usually have a 60–80% 5-year survival rate, which is decreased to 15–30% for patients with metastatic or recurrent disease. Consequently, chemotherapeutic treatments coupled with surgical techniques have been developed to treat OS.^4, 5, 6, 7, 8^ However, although the biology and genetics of OS have gained attention, the clinical outcomes of OS patients have not yet significantly improved.^1, 9, 10, 11, 12^ Thus, the improved identification of tractable candidates for OS patients is urgently required and may yield promising approaches to enhance the clinical management of OS.

MicroRNAs (miRNAs), which have emerged as post-transcriptional modulators of target genes, are endogenous small non-coding RNAs that have been described to have critical roles in genomic organization, biogenesis and other key cellular processes.^13, 14, 15, 16^ Currently, numerous studies have profiled miRNAs that act as oncogenes or tumour suppressors, thus suggesting that miRNAs may contribute to the renewed understanding of important diagnostic biomarkers and therapeutic targets for cancer.^15, 17, 18, 19^ Abnormal expression of miRNAs may lead to impairment of normal function. Recently, several miRNAs, such as miR-21, miR-34a, miR-143 and miR-382, have been demonstrated to be involved in the osteogenic differentiation and metastasis of OS.^20, 21, 22, 23, 24, 25^ However, the molecular mechanisms and specific roles of miRNAs in OS have not yet been clearly understood.

Cyclin-dependent kinases (CDKs), which are critical regulatory enzymes that drive cell cycle transition, are serine/threonine kinases characterized by their need for a separate subunit – a cyclin – to provide the essential domains for the enzymatic activity of the CDKs.^26, 27, 28^ CDKs have crucial roles in the control of cell cycle transition and have thus long been considered promising therapeutic targets for cancer therapies.^29, 30, 31^ The human genome encodes 26 serine/threonine protein kinases that form CDKs and CDK-like branches of the human kinome; of these, 21 are classified as CDKs.^32, 33^ Different CDK isoforms have key roles in cancer cell proliferation. Recent reports have shown that CDKs support the expression of inflammatory mediators.^34, 35^ CDK overexpression has been reported in various human cancers, such as gastric cancer, ovarian cancer, breast cancer, lung cancer and colorectal cancer, and has been correlated with cancer prognosis.^36, 37, 38, 39, 40^ An understanding of the biology of CDKs is critical for assessing the clinical results seen with CDK inhibitors, particularly with regard to the determination of their potential therapeutic windows and combination strategies. CDK14, also called PFTK1 (PFTAIRE protein kinase 1), is highly expressed in breast cancer, oesophageal cancer, lung cancer, liver cancer, gastric cancer, pancreatic cancer and ovarian cancer.^41, 42, 43, 44, 45, 46, 47, 48^ Although CDK14 has been implicated in cancer development and progression, its essential role in OS oncogenesis and progression remains unknown.

In the current study, we demonstrated that CDK14 was closely correlated with the overall survival (OS) and prognosis of OS patients. In addition, CDK14 was identified as a novel direct target and functional modulator of miR-216a in OS. We also demonstrated that miR-216a overexpression inhibited cell proliferation, invasion and migration, by using *in vivo* and *in vitro* approaches. Moreover, miR-216a expression was found to be downregulated in OS patients and negatively associated with CDK14 expression. Thus, miR-216a and CDK14 may be novel prognostic biomarkers and common therapeutic targets for treating OS.

## Results

### Expression of CDK14 and its correlation with clinical parameters in OS patients

**Figure 1:**
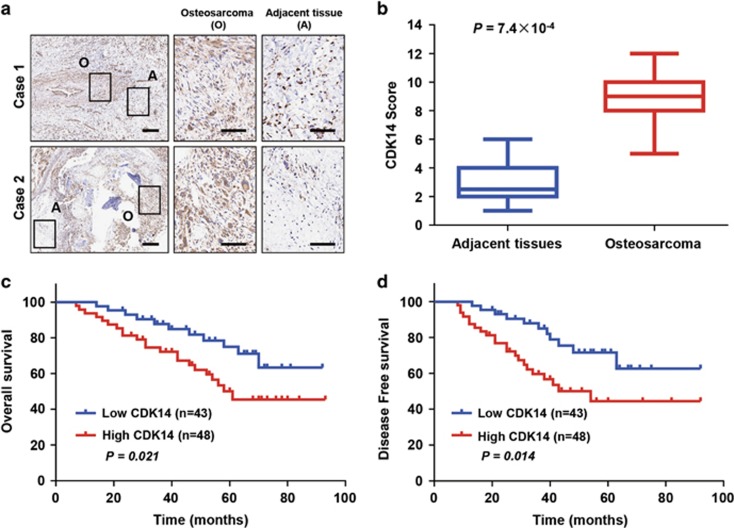
Expression of CDK14 and the correlation between CDK14 and clinical parameters in OS patients. (**a**) Representative IHC images of CDK14 expression in OS (O) tissues and adjacent (**a**) tissues. Scale bars: 500 *μ*m (left) and 100 *μ*m (middle and right). (**b**) CDK14 expression scores in OS tissues and matched adjacent normal tissues (*n*=91) were compared with the Mann–Whitney *U*-test. (**c**and **d**) Kaplan–Meier survival curves and log-rank tests were used to compare (**c**) OS and (**d**) DFS of the OS patients with low and high scores for CDK14

To identify the role of CDK14 in OS, we evaluated CDK14 expression in 91 OS samples and adjacent noncancerous tissues by using histopathologic assays (Figure 1a; Supplementary Figures 1 and 2). CDK14 expression was significantly upregulated in the OS tissues compared with the corresponding nontumour tissues (*P*=7.4 × 10^−4^; Figure 1b). To further investigate the clinical significance of CDK14, we examined the relationship between CDK14 levels and clinicopathological characteristics. The results showed that the level of CDK14 was closely correlated with tumour size and histological grade (Table 1). Moreover, Kaplan–Meier survival analysis indicated that patients with high levels of CDK14 had poorer OS (*P*=0.021) and disease-free survival (DFS; *P*=0.014) than those with low CDK14 expression (Figures 1c and d). Together, these results strongly suggested the importance of CDK14 in OS prognosis and metastasis.

### miR-216a inhibits CDK14 expression by directly targeting the 3′-UTR of CDK14

**Figure 2:**
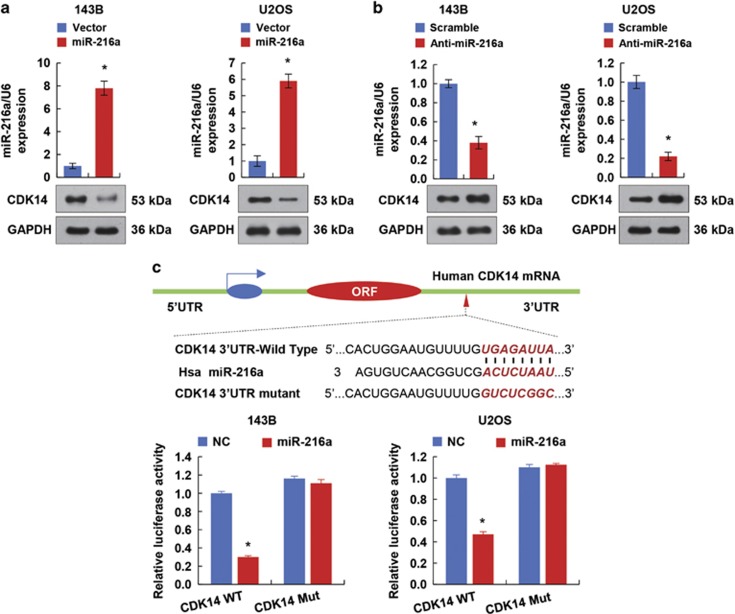
miR-216a suppresses the expression of CDK14 by targeting its 3′-UTR. (**a**and **b**) Immunoblot analysis of the indicated OS cell lines transfected with miR-216a or anti-miR-216a. The histograms under the immunoblots show the corresponding miR-216a mRNA expression levels. (**c**) miRNA luciferase reporter assays in 143B and U2OS cells co-transfected with wild-type or mutated CDK14 reporters and miR-216a. The top panel indicates wild-type and mutant forms of putative miR-216a target sequences in the 3′-UTR of CDK14. Bold and italicized fonts indicate putative miR-216a-binding sites in the 3′-UTR of human CDK14. Underlining indicates mutations introduced into the 3′-UTR of CDK14. Each bar represents the mean±S.D. of at least three independent experiments performed in triplicate (*P<0.05 *versus* corresponding control)

To identify the potential miRNA candidates that target CDK14, we used two target prediction programmes, miRanda and TargetScan, to predict the probability of a functional binding site. Several potential CDK14-targeting miRNAs were picked out, including miR-216a, miR-138, miR-205 and miR-455. Next, we predicted the free energy of the binding between the miRNAs and the CDK14 3′-UTR using the software at http://RNA.tbi.univie.ac.at/cgi-bin/RNAWebSuite/RNAcofold.cgi, and then carried out the western blot analysis to confirm the effect of the above-mentioned miRNAs on CDK14 expression in 293T cells (Supplementary Figure 3). Consistent with the results previously reported in breast cancer cell lines,^41^ miR-455 also inhibited the CDK14 expression in OS cell lines. Importantly, miR-216a had the optimal free energy and the most pronounced inhibitory effect on CDK14 expression. Thus, we hypothesize that miR-216a might exert an important role in OS. Indeed, according to western blot analysis, miR-216a significantly inhibited CDK14 expression in OS cell lines (Figure 2a; Supplementary Figure 3). In contrast, inhibition of miR-216a promoted the expression of CDK14 in the same OS cell lines (Figure 2b). Notably, miR-216a did not modulate the mRNA level of CDK14, thus suggesting that this regulation is post-transcriptional (Supplementary Figure 5).

We next transiently co-transfected 143B and U2OS cell lines with luciferase reporter constructs containing mutated or wild-type CDK14 3′-UTR and miR-216a or anti-miR-216a to evaluate whether CDK14 is a direct and specific target of miR-216a. miR-216a overexpression suppressed the CDK14 3′-UTR reporter activity, but did not affect the luciferase activity of the reporter in which the binding sites for miR-216a were mutated (Figure 2c). On the other hand, miR-216a inhibition upregulated the luciferase activity of the CDK14 3′-UTR reporter (Supplementary Figure 6). Therefore, these findings collectively suggested that miR-216a inhibits CDK14 production by directly targeting its 3′-UTR in OS cells.

### miR-216a suppresses OS cell proliferation, migration and invasion through the inhibition of CDK14 expression

**Figure 3:**
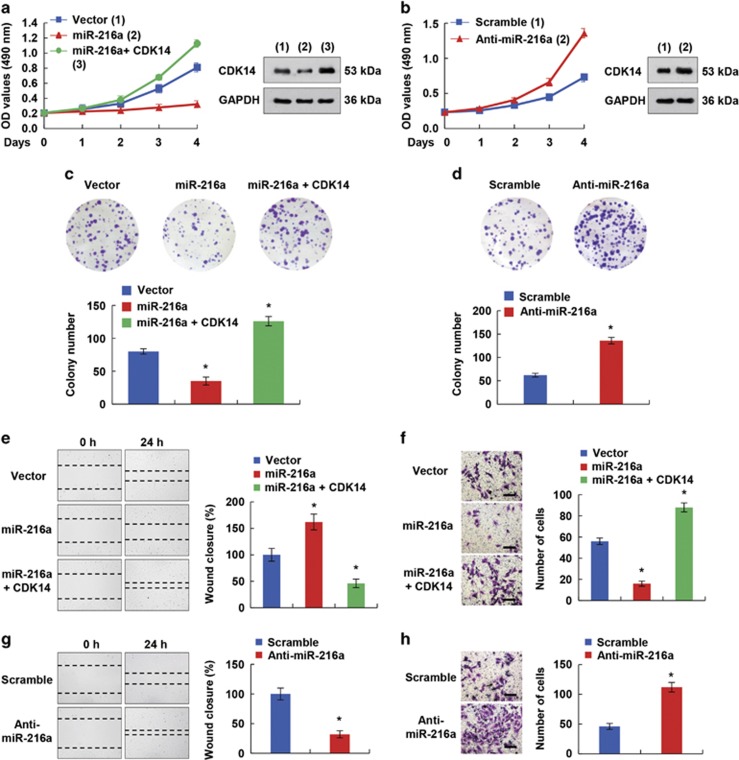
miR-216a suppresses cell proliferation, migration and invasion through the inhibition of CDK14 expression. (**a** and **b**) 143B cells expressing miR-216a or miR-216a and CDK14 (**a**) and 143B cells transfected with miR-216a inhibitor (**b**) were cultured in regular medium. At the specified times, cell numbers were determined with the CCK-8 assay. The representative immunoblot shows CDK14 expression. (**c** and **d**) 143B cells transfected with miR-216a (**c**) or miR-216a inhibitor (**d**) were plated and assayed for colony formation after 3 weeks. Representative images show colonies in plates (left panels). (**e** and **g**) Wound healing was conducted in 143B cells transfected with miR-216a or miR-216a and CDK14 (**e**) or miR-216a inhibitor (**g**). Cell migration was measured 24 h after the cell layers were scratched. Scale bar: 100 *μ*m. (**f** and **h**) Invasion of 143B cells transfected with miR-216a or miR-216a plus CDK14 (**f**) or miR-216a inhibitor (**h**) was evaluated using a Matrigel invasion chamber. The invaded cells were fixed and stained with crystal violet (**f** and **h** left images). Scale bar: 100 *μ*m. Each bar represents the mean±S.D. of at least three independent experiments performed in triplicate (*P<0.05 *versus* corresponding control)

We then evaluated whether miR-216a regulates phenotypes of OS cell lines, by using cell growth and colony formation assays. The cells were transfected with miR-216a and then used for cell growth analysis. In line with the above results, miR-216a overexpression suppressed the proliferative ability and colony formation of OS cells (Figures 3a and c). In addition, the introduction of CDK14 reversed the effect of miR-216a on cell proliferation (Figures 3a and c). In contrast, inhibition of miR-216a promoted the proliferation and colony formation of OS cells (Figures 3b and d). Hence, the above results demonstrated that miR-216a impaired cell proliferation though the inhibition of CDK14 expression.

Next, we investigated whether miR-216a had an effect on the migratory and invasive abilities of OS cells. Indeed, miR-216a overexpression inhibited cell migration, and re-expression of CDK14 impaired the migratory ability induced by miR-216a in wound-healing assays (Figure 3e). Similar results were also obtained with Matrigel invasion assays. Briefly, the results indicated that miR-216a overexpression markedly suppressed invasion of OS cell lines, whereas restoration of CDK14 reversed the effects of miR-216a (Figure 3f). Furthermore, knockdown of miR-216a, compared with a control treatment, promoted the migration and invasion of OS cells (Figures 3g and h), a result consistent with the findings above, thus indicating that CDK14 is a crucial mediator of miR-216a function in regulating OS metastasis.

### miR-216a/CDK14 axis regulates cell cycle progression

**Figure 4:**
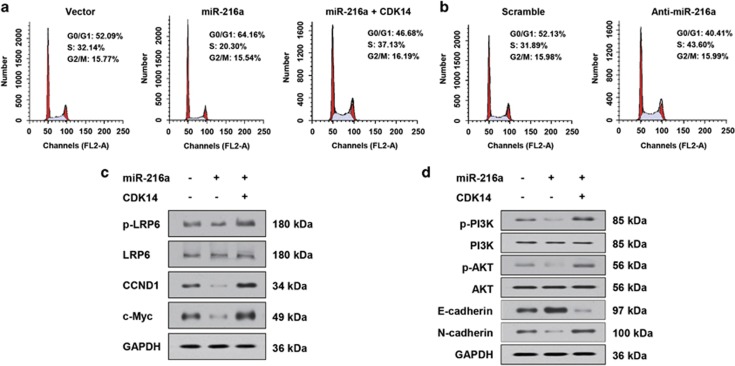
miR-216a suppresses cell cycle progression of OS cells. (**a** and **b**) Flow cytometry quantitation of cell cycle progress in 143B cells transfected with miR-216a or miR-216a and CDK14 (**a**) or miR-216a inhibitor (**b**). (**c** and **d)** Histogram of protein expression of the indicated genes in 143B cells transfected with miR-216a or miR-216a and CDK14

As miR-216a targets CDK14, a cell cycle CDK, to elucidate the mechanism how miR-216a suppresses OS cell growth, we investigated the effect of miR-216a on cell cycle distribution by flow cytometry analysis. Overexpression of miR-216a in 143B cells resulted in an increase in the proportion of cells in G0/G1 phase (from 52.09 to 64.16%) but a reduction in the proportion of cells in S phase (from 32.14 to 20.30% Figure 4a). In contrast, inhibition of miR-216a in 143B cells significantly reduced the proportion of cells in G0/G1 (52.13 to 40.41%) phases, which associated with an increase in proportion of cells in S phase (31.89 to 43.60% Figure 4b). These data suggest that miR-216a inhibits the G1/S transition in OS cells.

### miR-216a/CDK14 axis regulated cell migration and invasion via controlling lipoprotein receptor-related protein 6-mediated Wnt signalling pathway and PI3K/Akt pathway

It has been revealed that CDK14 mediates the phosphorylation of lipoprotein receptor-related protein 6 (LRP6), the co-receptor for Wnt ligands, thereby promoting Wnt signalling. In addition, knockdown of CDK14 inhibits the expression of p-PI3K and p-Akt in pancreatic cancer cells. Therefore, we investigated whether Wnt and PI3K/Akt signalling pathways were involved in miR-216a/CDK14-regulating cell invasion and migration. Our data showed that miR-216a decreased the phosphorylation level of the LRP6, as well as two key downstream targets of Wnt signalling pathway, CCND1 and c-Myc, while re-expression of CDK14 increased the levels of pLRP6, CCND1 and c-Myc (Figure 4c). Furthermore, miR-216a inhibited the phosphorylation of PI3K and Akt in OS cells and re-expression of CDK14 re-increased the phosphorylation levels of PI3K and Akt (Figure 4d). Taken together, these data collectively suggest that miR-216a/CDK14 axis regulated cell migration and invasion via controlling LRP6-mediated Wnt signalling pathway and PI3K/Akt pathway.

### miR-216a suppresses tumour initiation and metastasis of OS

**Figure 5:**
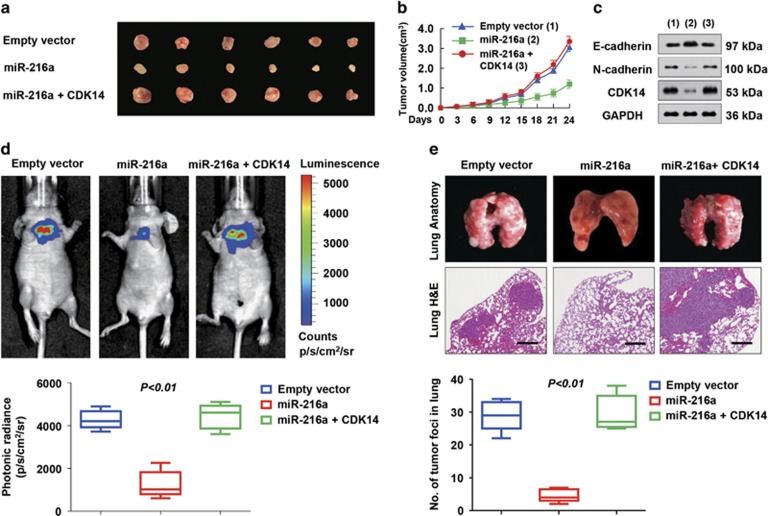
miR-216a suppresses tumour growth and metastasis of OS cell lines *in vivo*. (**a** and **b**) Stable 143B cells overexpressing miR-216a and miR-216a and CDK14 were injected into nude mice. At the indicate times, tumours were measured with Vernier calipers (mean±S.D.; *n*=6). (**c**) Immunoblot analysis of representative excised tumours in **a**. (**d**) Bioluminescence imaging of metastasis of OS cells in NOD-SCID mice at 30 days after intravenous injection of cells infected with PCDH control, PCDH-miR-216a or PCDH-miR-216a and CDK14 via the lateral tail vein. The luminescence signal is represented by an overlaid false-colour image with the signal intensity indicated by the scale. (**e**) Representative metastatic foci of lungs were subjected to anatomical and histological analyses. The data are shown as the mean±S.D. (*n*=6)

We further determined the *in vivo* phenotype of miR-216a expression by evaluating its effect on 143B cell growth in nude mice. Indeed, miR-216a overexpression significantly inhibited tumour growth (Figures 5a and b). In addition, tumours in mice inoculated with miR-216a-overexpressing 143B cells had decreased expression of CDK14 and EMT markers (Figure 5c). Moreover, tumours in mice formed by miR-216a plus CDK14-overexpressing 143B cells showed a reversal of the miR-216a effect on tumour growth (Figures 5a and b).

Next, we investigated the effect of miR-216a on metastasis. The results revealed that the miR-216a-expressing group, compared with the control group, displayed a more significant decrease in the metastatic burden in the lungs. Moreover, the photonic radiance intensity of the lungs in the miR-216a-expressing group showed similar results (Figure 5d). In contrast, the miR-216a plus CDK14 group displayed impaired miR-216a expression (Figure 5d). Anatomic and histologic analysis of the lungs also confirmed the metastatic foci (Figure 5e). Briefly, the miR-216a-expressing group had a lower number of tumour foci in the lungs than the control group, whereas the miR-216a plus CDK14 group displayed reversal of the effects of miR-216a on the metastatic foci. In addition, the miR-216a-expressing group had better survival probability (*P*=0.002) than the controls according to the Kaplan–Meier survival analysis (Supplementary Figure 7). Together, these results strongly supported the role of miR-216a as a suppressor of tumour dissemination.

### Expression of miR-216a and CDK14 and the correlation between miR-216a and CDK14 in OS samples

**Figure 6:**
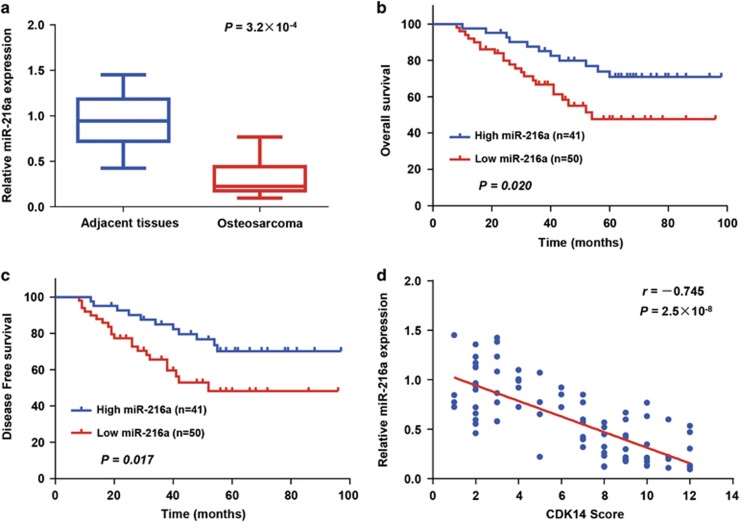
Expression of miR-216a and its correlation with CDK14 in OS patients. (**a**) Expression of miR-216a in OS tissues and matched adjacent normal tissues (*n*=91) was compared using the Mann–Whitney *U*-test. U6 small nuclear RNA was used as the internal control. (**b** and **c**) Kaplan–Meier survival curves and log-rank tests were used to compare (**b**) OS and (**c**) DFS of OS patients with low and high expression levels of miR-216a. (**d**) The relationship between miR-216a and CDK14 expression was assessed by Spearman’s rank correlation analysis in the OS samples. The symbols represent individual samples

To assess the clinical significance of miR-216a, we evaluated its level in 91 OS samples and matched adjacent nontumour tissues, using quantitative reverse-transcription PCR (qRT-PCR). On the basis of the qRT-PCR results, miR-216a expression in OS patients was significantly decreased (*P*=3.2 × 10^−4^; Figure 6a). To further investigate the clinical significance of CDK14, we examined the relationship between miR-216a expression and clinicopathological characteristics. The results showed that the level of miR-216a expression was closely correlated with tumour size and histological grade (Supplementary Table 2). In addition, according to the Kaplan–Meier survival analysis, patients with high miR-216a expression levels had better OS (*P*=0.020) and DFS (*P*=0.017) than did patients with low expression levels of miR-216a, thus suggesting that miR-216a is a predictor of better clinical outcomes (Figures 6b and c). Furthermore, in agreement with miR-216a inhibition of CDK14 protein expression in cultured cells (Figure 2a; Supplementary Figure 3), miR-216a expression was negatively associated with CDK14 protein expression in the OS samples (*P*=2.5 × 10^−8^, *r*=−0.745; Figure 6d). Overall, these findings strongly indicated the crucial role of miR-216a and CDK14 in the prognosis of OS.

## Discussion

To date, accumulating studies have implicated miRNAs as critical components in modulating various biological processes and cellular functions.^18, 49, 50, 51^ Aberrant expression of miRNAs is closely correlated with proliferation, invasion, metastasis and prognosis in various cancers.^17, 52, 53, 54^ Therefore, better knowledge of miRNAs concerning gene networks may provide novel mechanistic insights into oncogenesis and facilitate current therapies for cancer. Studies have revealed that miR-216a is one of the miRNAs that is downregulated in various types of solid tumours. In colorectal cancer, miR-216a suppresses tumour metastasis an invasion through downregulation of KIAA1199/CEMIP.^55^ Besides, miR-216a decreases MALAT1 and JAK2 expression in pancreatic cancer cells.^56, 57, 58^ In addition, miR-216a also exerts its function as a tumour suppressor in prostate cancer, lung cancer and liver cancer.^58, 59, 60^ Moreover, for oral squamous cell carcinoma, miR-216a inhibits the growth and metastasis by targeting eukaryotic translation initiation factor 4B.^61^ However, investigations on the tumour suppressive role of miR-216a in OS are still lacking. In this study, we found that miR-216a exerts an inhibitory effect on OS cell proliferation, migration and invasion by directly targeting CDK14. In addition, the expression of miR-216a in OS patients was significantly decreased. Moreover, patients with high miR-216a expression levels had better OS and DFS, thus suggesting that miR-216a has a predictive and prognostic role in OS.

CDK14, a cell division cycle 2-related serine/threonine protein kinase, interacts with Cyclin D3 and acts as an essential regulator of CDK-cyclins (CCNs) and cell cycle progression.^62, 63, 64^ In the present study, we show that the miR-216a/CDK14 axis modulates cell cycle progression at G1 phase in OS cells. Emerging information on the molecular mechanisms of CDK14 also indicates that CDK14 mediates the phosphorylation of cell cycle-dependent low-density LRP6, thereby promoting Wnt signalling.^62, 65^ In addition, knockdown of CDK14 inhibited the expression of PI3K and Akt phosphorylation in pancreatic cancer cells.^47^ In this study, we demonstrated that miR-216a/CDK14 axis also regulated the phosphorylation of LRP6 and the expression of other downstream genes in Wnt signalling in OS cells. Besides, miR-216a/CDK14 axis also exerted an important role in PI3K and Akt phosphorylation in OS cells. Moreover, abnormal functions of CDK proteins contribute to promote lung, breast, colorectal, ovarian and various other cancers, thereby suggesting that CDKs might be potential attractive targets for therapeutic intervention.^27, 28, 30, 66^ CDK14 expression directly increases with cancer progression. Importantly, CDK14 has been correlated with oncogenesis-associated cellular properties such as invasiveness and motility. EMT, a cellular process during which epithelial polarized cells become motile mesenchymal-appearing cells, has an important role in tumour metastasis and invasion. The E-cadherin and N-cadherin are two important markers for the EMT. E-cadherin and N-cadherin are closely associated with invasion and metastasis. In tumour, the forced expression of E-cadherin inhibited cancer metastasis and the mutation of E-cadherin would promote cellular invasion, motility and metastasis, and suppress cellular adhesion.^67, 68^ In addition, it has been reported that vimentin was considered as the mechanical transducer between the nucleus and cell surface, and thus controls cell migration through cell adhesion stability regulation.^69^ Studies have revealed that knockdown of CDK14 in cancer cells promoted E-cadherin expression, thus suppressing EMT.^44, 47, 70^ However, the relationship between N-cadherin and CDK14 has not been fully elucidated. As miR-216a/CDK14 axis modulated OS tumour invasiveness, we thus sought to explore the effects of miR-216a/CDK14 axis on the E-cadherin and N-cadherin production in mice. As expected, miR-216a inhibited the expression of the N-cadherin and promoted E-cadherin expression, suggesting that miR-216a/CDK14 axis is closely correlated with the EMT in OS.

To date, studies have indicated that CDK14 depletion impairs tumour angiogenesis. The expression of CDK14 is upregulated in lung cancer, whereas the inhibition of CDK14 expression leads to the suppression of non-small-cell lung cancer proliferation and invasion through the Wnt/*β*-catenin signalling pathway.^44^ A recent study has revealed that miR-455 inhibits breast cancer cell proliferation by targeting CDK14,^41^ as was also demonstrated in OS cells in our study. However, the exact role of CDK14 and the miRNA in regulating CDK14 expression in OS has not been fully elucidated. Conceivably, CDK14 downregulation might be a promising molecular strategy for OS therapy. In this study, we found that high expression levels of CDK14 were more frequent in OS tissues and patients with high levels of CDK14 had shorter OS and DFS, while the suppressive effect of miR-216a led to diminished cell proliferation, migration and invasion *in vitro* and *in vivo*, revealing that the miR-216a/CDK14 axis may a new and promising target for the prevention of OS tumourigenesis and metastasis.

qRT-PCR analysis indicates that miR-216a does not modulate CDK14 mRNA expression level, but significantly inhibits CDK14 protein expression in cultured cells, suggesting that the regulation of CDK14 by miR-216a is post-transcriptional. In clinical samples, CDK14 expression was detected by immunostaining the CDK14 protein with the CDK14-specific antibody. And our clinical analysis data revealed a negative relationship between miR-216a expression and CDK14 protein expression in the OS samples, which was consistent with the results gained in the cell lines.

Collectively, our results demonstrated that CDK14 is a novel independent marker that predicts the clinical outcomes of OS. miR-216a can suppress the proliferation, migration and invasion in OS cells by targeting CDK14 expression. The expression of miR-216a was downregulated in OS patients and negatively correlated with that of CDK14, thus suggesting that the miR-216a/CDK14 axis may be an ideal predictor of clinical outcomes in OS.

## Materials and methods

### Patients and specimens

This study was approved by the Institutional Review Committee of the General Hospital of the People’s Liberation Army (Beijing, China) and was conducted with informed consent of the patients. A total of 91 conventional OS and adjacent noncancerous tissues, evaluated on the basis of accepted pathological and radiological criteria, were used in the study. Clinical information was collected from the patient records. The OS was defined as the time elapsed from surgery to death. The follow-up information of the patients was updated every month. The specimens were divided into two portions: one portion was immediately snap-frozen in liquid nitrogen and stored at −80 °C until RNA extraction, and the other portion was used for histopathologic assessment. The clinical and demographic characteristics of the study population are given in Supplementary Table 1.

### Plasmids and reagents

Wild-type or mutant promoter-containing luciferase reporters were generated through the insertion of PCR-amplified promoter fragments from genomic DNA into the pGL4-Basic vector (Promega, Madison, WI, USA). The primer sequences are displayed in Table 2. Eukaryotic expression vectors encoding FLAG fusion proteins tagged at the amino terminus were constructed by inserting PCR-amplified fragments into pcDNA3 (Invitrogen, Waltham, MA, USA). To introduce mutations into the seed sequences of predicted miR-216a target sites within the 3′-UTR of CDK14, recombinant PCR was performed using the primers mentioned (Table 2). Lentiviruses were produced by co-transfection of HEK293T cells with recombinant lentiviral vectors and pPACK Packaging Plasmid Mix (System Biosciences, Mountain View, CA, USA) using MegaTran reagent (Origene, Rockville, MD, USA). Lentiviruses were collected 48 h after transfection and added to the medium of the target cells with 8 *μ*g/ml polybrene (Sigma-Aldrich, St. Louis, MO, USA). Stable cell lines were selected with 1 *μ*g/ml puromycin for ~2 months. Pooled clones or individual clones were screened by standard immunoblotting protocols and produced similar results.

Anti-CDK14 (HPA015267, SAB2107669) and anti-E-cadherin (SAB4503751) antibodies were purchased from Sigma-Aldrich. Anti-GAPDH (ab37168), anti-CCND1 (ab134175), anti-c-Myc (ab11917), anti-PI3K p85 (ab86714), anti-phosphor-PI3K p85 (ab182651), anti-AKT (ab126811), anti-phosphor-AKT (ab183758) and anti-N-cadherin (ab12221) antibodies were purchased from Abcam (Cambridge, MA, USA). LRP (C5C7) and anti-phosphor-LRP6 (Ser1490) antibodies were purchased from Cell Signalling Technology (Danvers, MA, USA).

### Cell culture and transfection

U2OS and 143B cell lines were purchased from the American Type Culture Collection (Manassas, VA, USA) and had been tested for mycoplasma contamination. The cells were routinely cultured in Dulbecco’s modified Eagle’s medium (DMEM) with high glucose supplemented with 10% fetal calf serum, 100 IU/ml penicillin and 100 *μ*g/ml streptomycin at 37 °C in an atmosphere with 5% CO_2_. For transfection, the cells were seeded in 6- or 24-well plates with the indicated plasmids using Lipofectamine 2000 (Invitrogen) according to the manufacturer’s instructions. The miRNA mimics were transfected into the cells using FuGENE HD (Promega) according to the manufacturer’s protocol. The miRNA inhibitors (Ambion, Grand Island, NY, USA) were transfected at a concentration of 50 nM.

### RNA extraction and quantitative reverse-transcription PCR

Total RNA was extracted and reverse-transcribed into cDNA using an RNeasy Mini kit (Qiagen, Valencia, CA, USA) according to the manufacturer’s instructions. RNA quality was checked using an Agilent 2100 Bioanalyzer (Santa Clara, CA, USA) and rated according to the RNA integrity number (RIN). RIN values range from 1 to 10 for totally degraded to intact RNA, with values >7 considered to be acceptable integrity for qRT-PCR gene expression examination. TaqMan miRNA qRT-PCR (Applied Biosystems, Foster City, CA, USA) was used to detect and quantify miRNA expression as previously described.^71^ The relative expression level of the miRNA was calculated using the comparative Ct method. Universal small nuclear RNA U6 (RNU6B) was used as the endogenous control for the miRNAs. The sequences of the primers used for qRT-PCR analysis are listed in Table 2.

### Luciferase assay

The cells were seeded in 24-well plates at 60% confluence. Reporter constructs containing the wild-type or mutant 3′-UTR of CDK14 were co-transfected with miR-216a into cells with Lipofectamine 2000 reagent, according to the manufacturer’s protocol. After 48 h, the cells were collected and examined for *β*-galactosidase and luciferase activities as previously described.^72^

### Western blotting

Total protein extracts were prepared for western blot analysis as previously described. The membranes were incubated with antibodies to CDK14 (1:1000 dilution), FLAG-HRP (1:3000 dilution) and *β*-actin (1:500 dilution). The immunocomplexes were visualized via chemiluminescence using an ECL kit (Amersham Biosciences, Piscataway, NJ, USA).

### Cell invasion assays

Matrigel invasion chambers (BD Biosciences, San Jose, CA, USA) were used to measure cell invasion according to the manufacturer’s instructions. Briefly, cells were placed on the upper surface of the Transwell inserts. After 24 h, the invasive cells were fixed with 4% paraformaldehyde and stained with 0.5% crystal violet. The number of invasive cells was counted in five randomly selected microscopic views and photographed.

### Wound-healing assays

Cells were seeded in six-well plates at 70% confluence in culture medium for wound-healing assays. After 24 h, the confluent cellular monolayer was scratched with a fine pipette tip. For migration, the rate of wound closure was observed at the indicated times using a microscope.

### Anchorage-dependent and anchorage-independent growth assays

Cell proliferation was assessed by using a CCK-8 Kit (Dojindo Laboratories, Kumamoto, Japan) according to the manufacturer’s instructions. To analyse anchorage-independent growth, transfected cells were seeded in 96-well plates and examined at 0, 24, 48, 72 and 96 h as previously described.^73^

### Cell cycle assay

The OS cells were fixed with 70% ethanol at −20 °C for at least 24 h. After washing twice with the ice-cold PBS, the cells were incubated with RNase A (1 mg/ml) at room temperature for 20 min. Cells were then labelled in PBS with propidium iodide (50 mg/ml) (Becton-Dickinson). The cell cycle were examined using a Becton-Dickinson flow cytometer BD FACScan System along with Cell Quest acquisition. Gating was set to exclude the cell doublets, cell debris and cell clumps.

### Animal experiments

The animal studies were performed in accordance with protocols approved by the Institutional Animal Care and Use Committee at the General Hospital of the People’s Liberation Army. Approximately 1 × 10^7^ 143B cells were injected into 6-week-old BALB/c mice. For the tumour growth model, cells labelled with firefly luciferase and stably transfected with the pCDH control vector, pCDH-miR-216a or PCDH-miR-216a and CDK14 were subcutaneously injected. Tumour growth was determined by caliper measurements. Tumour volume was calculated according to the following formula: volume=(longest diameter × shortest diameter^2^)/2. Excised tumours were weighed, and portions were frozen in liquid nitrogen or fixed in 4% paraformaldehyde for further study. For *in vivo* lung metastasis study, 1 × 10^6^ 143B cells labelled with firefly luciferase carrying indicated constructs were injected into the lateral tail vein of BALB/c female mice. The animals were imaged on the day 35 using the IVIS200 imaging system (Xenogen Corporation, Alameda, CA, USA). The mice were then killed and the lung was weighted and fixed with 4% paraformaldehyde for further study.

### Histopathologic assessment

For histopathologic assays, tissues were fixed in 4% buffered paraformaldehyde for 48 h and subsequently decalcified with buffered EDTA (20% EDTA, pH 7.4). The tissues were embedded in paraffin, sectioned and stained with haematoxylin–eosin.

For immunohistochemistry (IHC) assays, briefly, the sections were pre-treated for 10 min with trypsin (0.05%) before treatment with 3% (vol/vol) H_2_O_2_ for 15 min. Then the sections were then blocked at room temperature for 1 h with 10% goat serum. After washing with PBS, anti-CDK14 antibody (1:50 dilution) was applied to the sections, and the sections were incubated overnight at 4 °C. The sections were then washed with PBS and incubated with biotinylated secondary antibody for 15 min using a Histostain Plus kit (Invitrogen, Carlsbad, CA, USA). The sections were washed and incubated with 3, 3′-diaminobenzidine substrate for 2 min.

IHC staining was detected by two pathologists blinded to the origin of the specimen using light microscopy. *H*-score method that combines the values of immunoreaction intensity and the percentage of cells stained was applied to determine the total immunohistochemical scoring as previously described.^72^ Briefly, *H*-score was achieved by multiplying the percentage of weakly stained cells (times 1), the percentage of moderately stained cells (times 2) and the percentage of strongly stained cells (times 3). Score ≤2.1 was defined as low score and score between 2.1 and 3 was defined as high score.

### Statistical analysis

Survival analysis was performed using the Kaplan–Meier method, and differences in survival curves were evaluated by the log-rank test. The Cox regression model was used to perform univariate and multivariate analyses. qRT-PCR data were evaluated using one-way ANOVA with Tukey’s *post hoc* test. Correlation was examined through Pearson’s *χ*2 analysis using GraphPad PRISM 6 (GraphPad, San Diego, CA, USA). All statistical tests were two-sided. Statistical calculations were performed using SPSS 17.0 (Chicago, IL, USA). All *in vitro* experiments were performed in triplicate and were repeated three times. The data are presented as the means±S.D. *P*<0.05 was considered statistically significant.

## Figures and Tables

**Table 1:** Associations between CDK14 expression and clinicopathological characteristics

**Characteristics**	***n***	**CDK14 expression**		***P***
		**High (*n*, %)**	**Low (*n*, %)**	
*Gender*				
Male	51	26 (51.0)	25 (49.0)	0.571
Female	40	18 (45.0)	22 (55.0)	
				
*Tumour size (cm)*				
>7	49	32 (65.3)	17 (34.7)	0.002**
≤7	42	14 (33.3)	28 (66.7)	
				
*Location*				
Distal femur	48	26 (54.2)	22 (45.8)	0.903
Proximal tibia	26	14 (53.8)	12 (46.2)	
Proximal humerus	11	5 (45.5)	6 (54.5)	
Proximal femur	4	3 (75.0)	1 (25.0)	
Others	2	1 (50.0)	1 (50.0)	
				
*TNM stage*				
I	44	12 (27.3)	32 (72.7)	2.209 × 10^−4^ **
II/III	47	31 (66.0)	16 (34.0)	
				
*Relapse*				
Yes	9	5 (55.6)	4 (44.4)	0.015*
No	82	16 (19.5)	66 (80.5)	
				
*Metastasis*				
Lung	34	22 (64.7)	12 (35.3)	0.013*
Others	2	1 (50.0)	1 (50.0)	
No	55	18 (32.7)	37 (67.3)	

*P*-values were calculated by Pearson’s *χ*^2^-test

*P<0.05

**P<0.01

**Table 2:** Primer sequences of oligonucleotides

**Name**	**Forward (5**′**→3**′**)**	**Reverse (5**′**→3**′**)**
*Primer sequences for real-time quantitative RT-PCR*		
CDK14	CAAACCCCTGGACACAATTCCTG	CGAGCTGGGGCTGGAGTGCCG
LRP6	ACAAAAGCTTTATTGGGCAGATGC	GGAGAGAAGATGTCAGAATGGATTT
CCND1	CTAAGATGAAGGAGACCATCCC	AAGGTCTGCGCGTGTTTGCGGAT
c-Myc	CAGGACTGTATGTGGAGCGGCTT	GCGAGCTGCTGTCGTTGAGAGGG
PI3K	CAAGTATATTTTAAAAGTGTGTGG	GATGTTTCTCCATTCATATATGGTG
AKT	CAACTCAGGGGCTGAAGAGATG	ACACACTCACCGAGAACCGCG
*β*-actin	ATCACCATTGGCAATGAGCG	TTGAAGGTAGTTTCGTGGAT
		
*Primers sequences for PCR*		
CDK14 3′-UTR	CTTGGAAATAACTGCACATTTATATA	ATTAGATGTTGACAAGACCCAGAC
CDK14 3′-UTR Mut	CACTGGAATGTTTTGGTCTCGGC	GTGACCTTACAAAACCAGAGCCG
CDK14	ATGTGTGACCTCATTGAGCCGC	TCAGTGCTTGCTGTTTGATAGAC
