# Supplementary material for: miR-216a inhibits osteosarcoma cell proliferation, invasion and metastasis by targeting CDK14
Source: Cell Death Dis. 2017 Oct 12;8(10):e3103–. doi: 10.1038/cddis.2017.499 (PMC5682665; doi:10.1038/cddis.2017.499)
Supplement: Supplementary Table 1 [file cddis2017499x1.doc]

**Supplementary Table 1 Clinicopathological characteristics of osteosarcoma patients**

| **Characteristics** | **n (%) *Median (range)** |
| --- | --- |
| Age  Gender  Male  Female  Tumor size (cm)  ＞ 7  ≤ 7  Location  Distal femur  Proximal tibia  Proximal humerus  Proximal femur  Others  TNM Stage  Ⅰ  Ⅱ/Ⅲ  Relapse  Yes  No  Metastasis  Lung  Others  No | 16.12 (6.63-18.37)*****  51 (56.0%)  40 (44.0%)  49 (53.8%)  42 (46.2%)  48 (52.7%)  26 (28.6%)  11 (12.1%)  4 (4.4%)  2 (2.2%)  44 (48.4%)  47 (51.6%)  9 (9.9%)  82 (90.1%)    34 (37.4%)  2 (2.2%)  55 (60.4%) |
