# Supplementary material for: miR-216a inhibits osteosarcoma cell proliferation, invasion and metastasis by targeting CDK14
Source: Cell Death Dis. 2017 Oct 12;8(10):e3103–. doi: 10.1038/cddis.2017.499 (PMC5682665; doi:10.1038/cddis.2017.499)
Supplement: Supplementary Table 2 [file cddis2017499x2.doc]

**Supplementary Table 2**

**Associations between miR-216a expression and clinicopathological characteristics**

| **Characteristics** | **n** | **miR-216a expression** | | **P** |
| --- | --- | --- | --- | --- |
|  |  | **High (n, %) Low (n, %)** | |  |
| Gender  Male  Female  Tumor size (cm)  ＞ 7  ≤ 7  Location  Distal femur  Proximal tibia  Proximal humerus  Proximal femur  Others  TNM Stage  Ⅰ  Ⅱ/Ⅲ  Relapse  Yes  No  Metastasis  Lung  Others  No | 51  40  49  42  48  26  11  4  2  44  47  9  82    34  2  55 | 20 (39.2%)  15 (37.5%)  14 (28.6%)  29 (69.0%)  20 (41.7%)  11 (42.3%)  7 (63.6%)  2 (50.0%)  1 (50.0%)  29 (65.9%)  19 (40.4%)  3 (33.3%)  60 (73.2%)  9 (26.5%)  1 (50.0%)  34 (61.8%) | 31 (60.8%)  25 (62.5%)  35 (71.4%)  13 (31.0%)  28 (58.3%)  15 (57.7%)  4 (36.4%)  2 (50.0%)  1 (50.0%)  15 (34.1%)  28 (59.6%)  6 (66.7%)  22 (26.8%)  25 (73.5%)  1 (50.0%)  21 (38.2%) | 0.867  1.155×10^-4^**  0.755  0.015*  0.014*  0.005* |

*P*-values were calculated by Pearson’s Chi-Square test.

**P* < 0.05, ***P* < 0.01
