# Supplementary material for: miR-216a inhibits osteosarcoma cell proliferation, invasion and metastasis by targeting CDK14
Source: Cell Death Dis. 2017 Oct 12;8(10):e3103–. doi: 10.1038/cddis.2017.499 (PMC5682665; doi:10.1038/cddis.2017.499)
Supplement: Supplementary Table 3 [file cddis2017499x3.doc]

**Supplementary Table 3**

**The raw mean Ct from the qPCR of the miR-216a expression levels in samples**

|  | Adjacent tissues | Osteosarcoma | 143B | U2OS |
| --- | --- | --- | --- | --- |
| Ct | 25 | 29 | 22 | 20 |
