# Supplementary figures and images for: miR-216a inhibits osteosarcoma cell proliferation, invasion and metastasis by targeting CDK14
Source: Cell Death Dis. 2017 Oct 12;8(10):e3103–. doi: 10.1038/cddis.2017.499 (PMC5682665; doi:10.1038/cddis.2017.499)

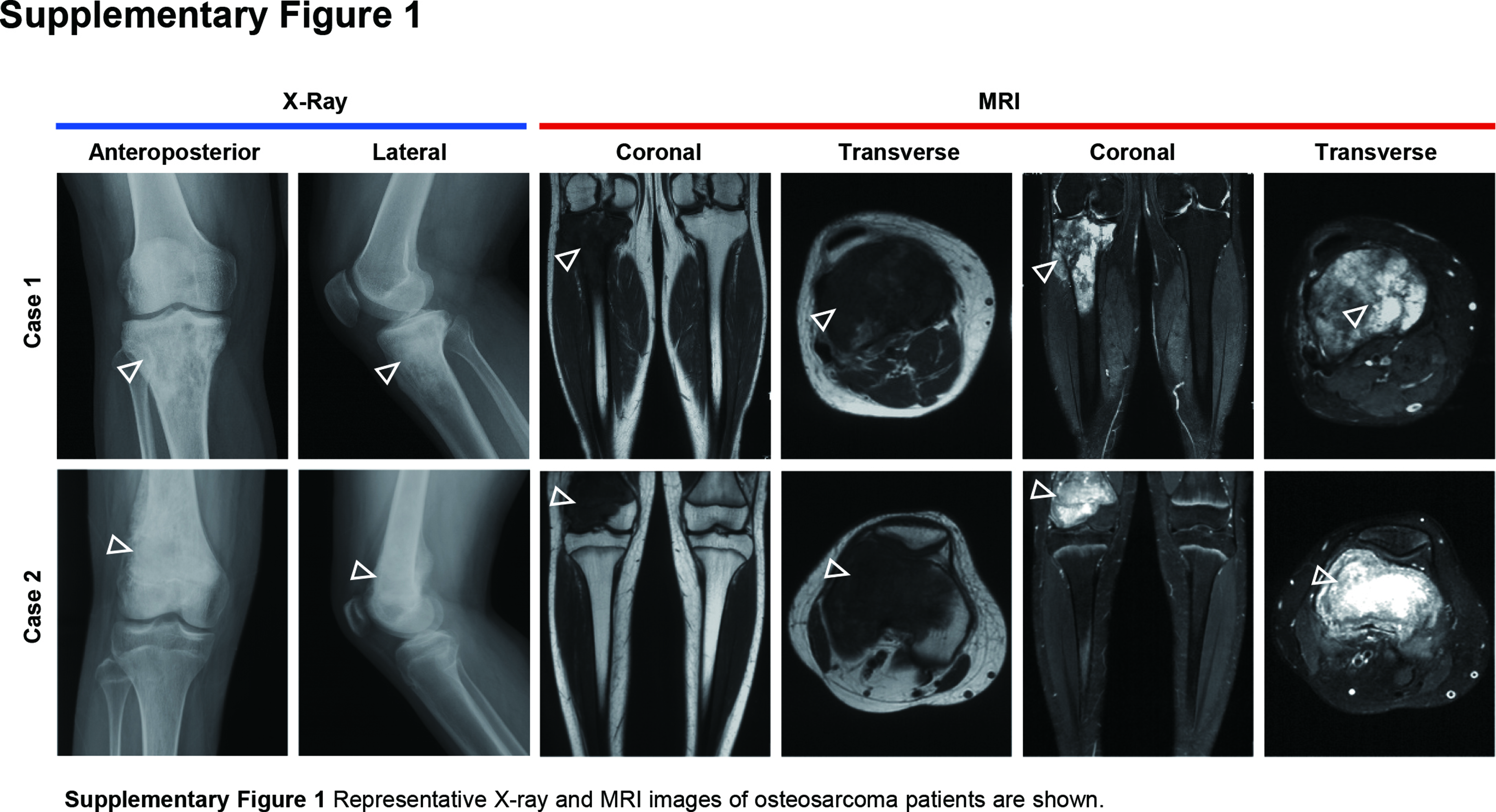

Supplement: Supplementary Figure 1 [file cddis2017499x4.tif]

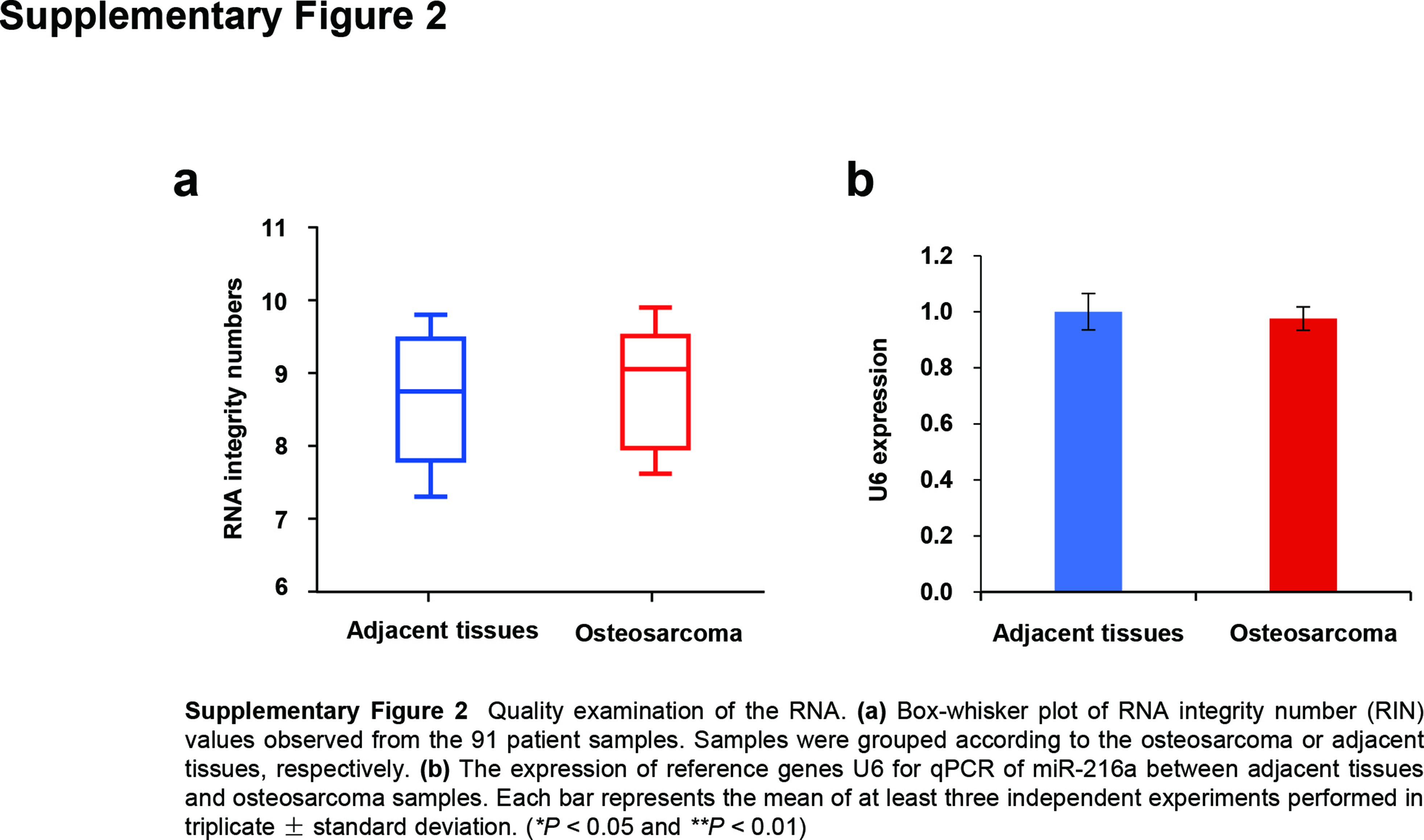

Supplement: Supplementary Figure 2 [file cddis2017499x5.tif]

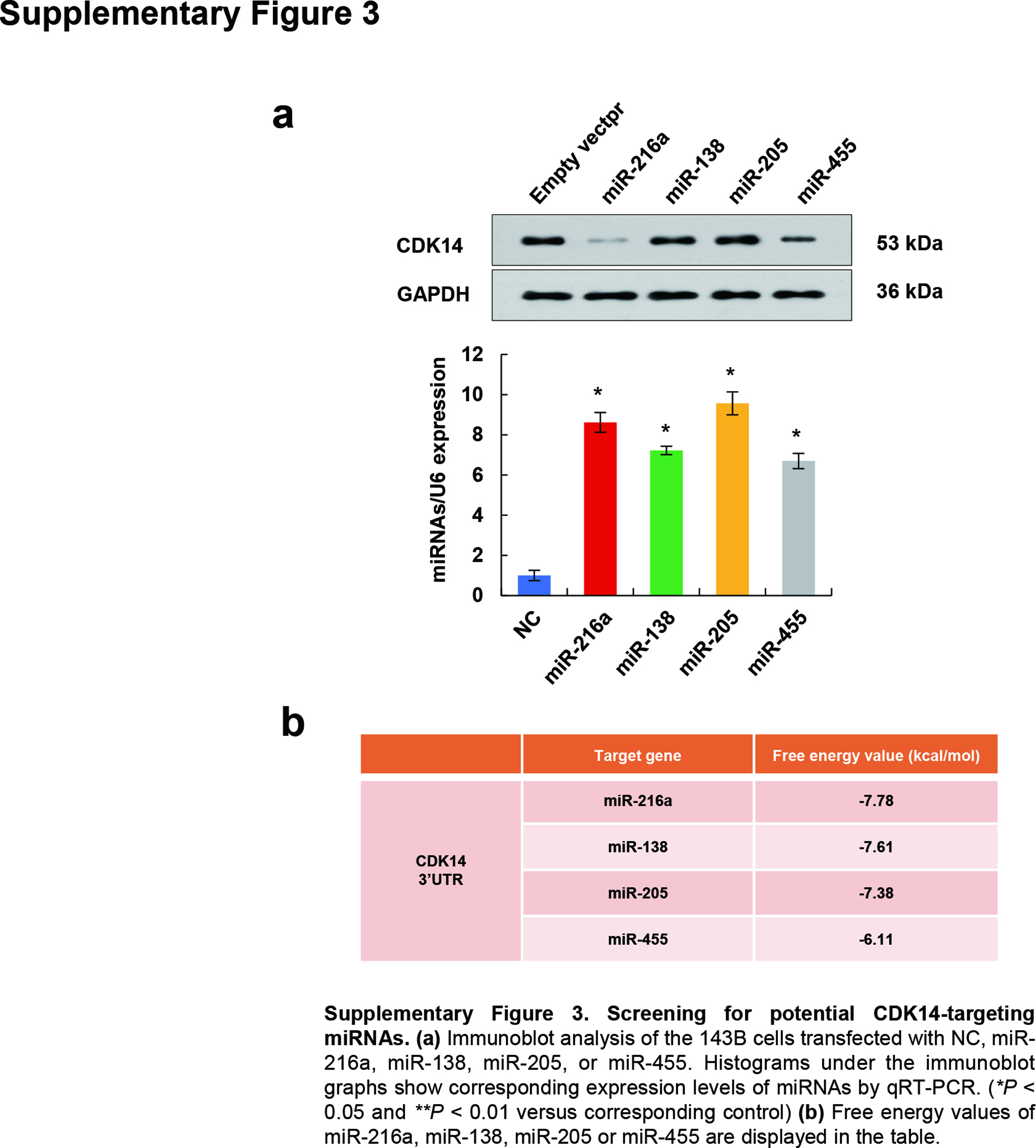

Supplement: Supplementary Figure 3 [file cddis2017499x6.tif]

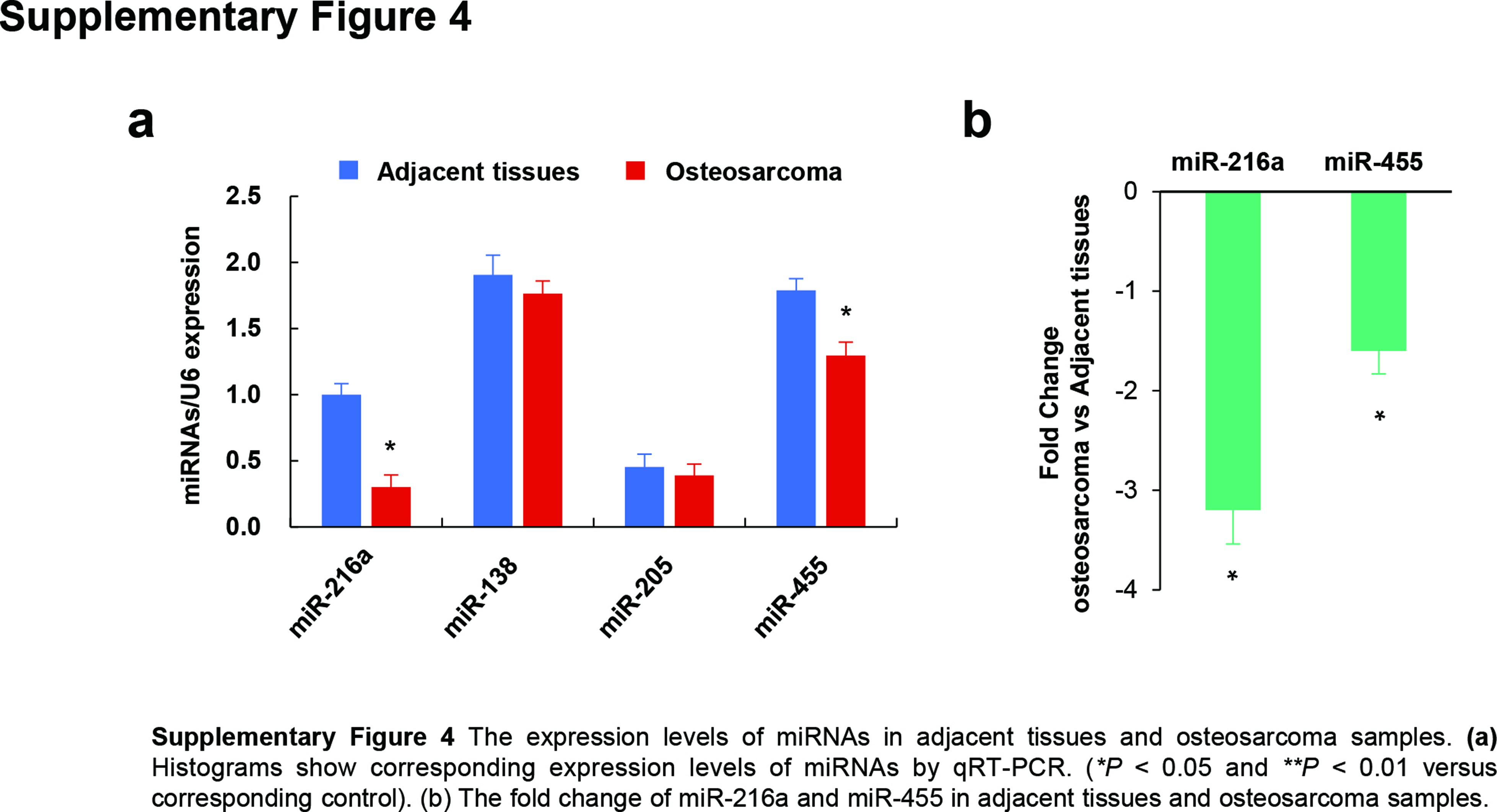

Supplement: Supplementary Figure 4 [file cddis2017499x7.tif]

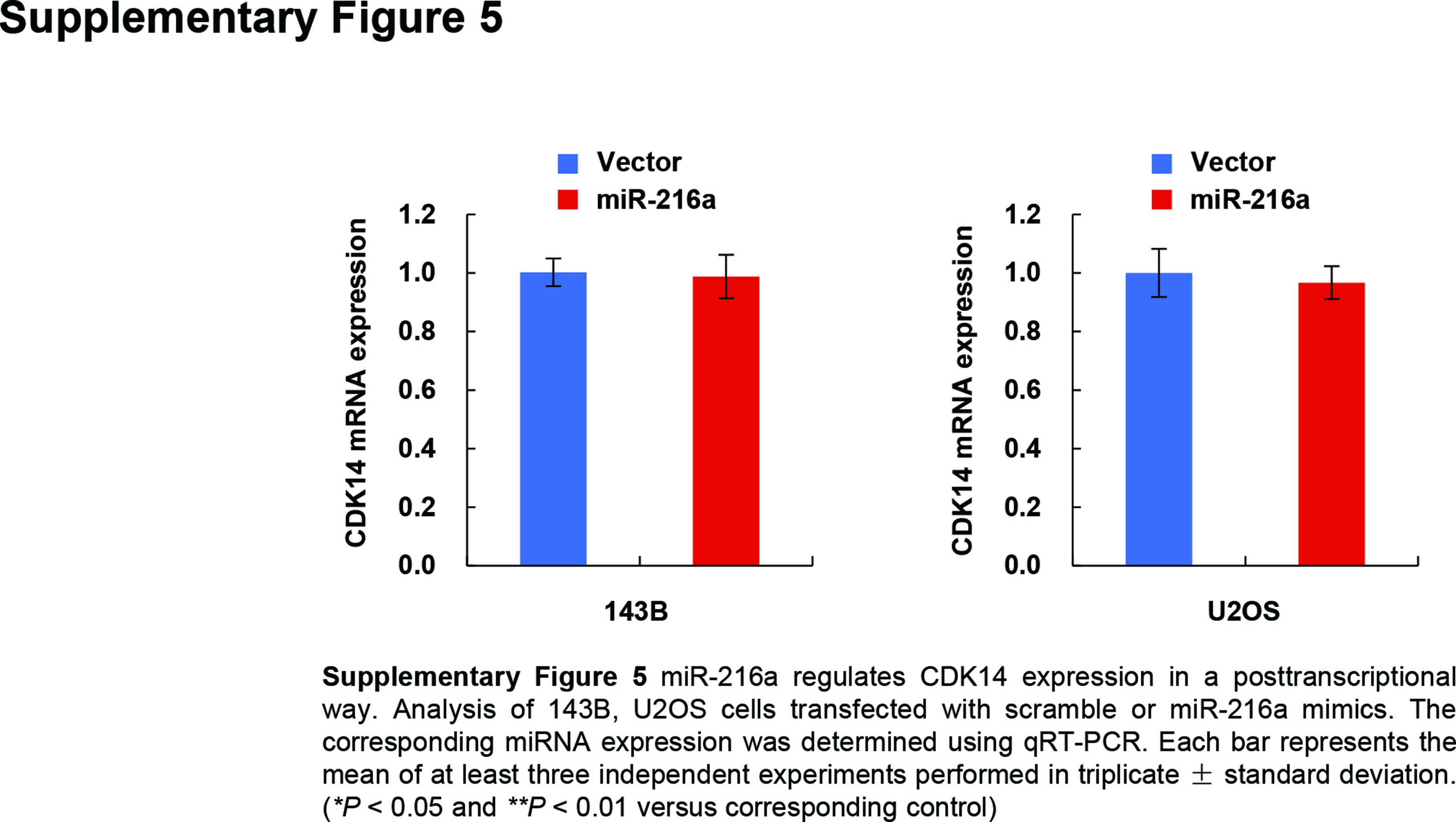

Supplement: Supplementary Figure 5 [file cddis2017499x8.tif]

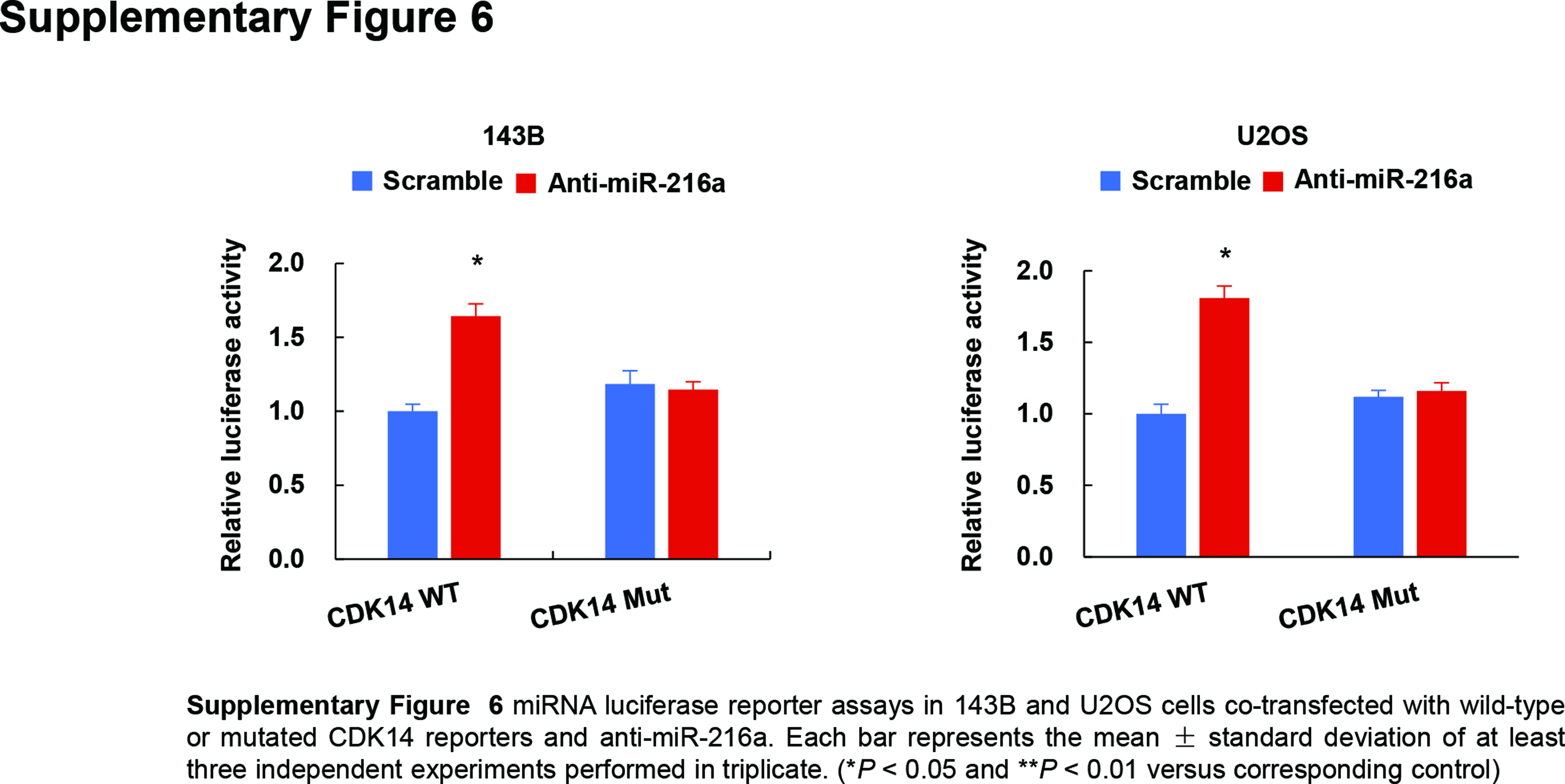

Supplement: Supplementary Figure 6 [file cddis2017499x9.tif]

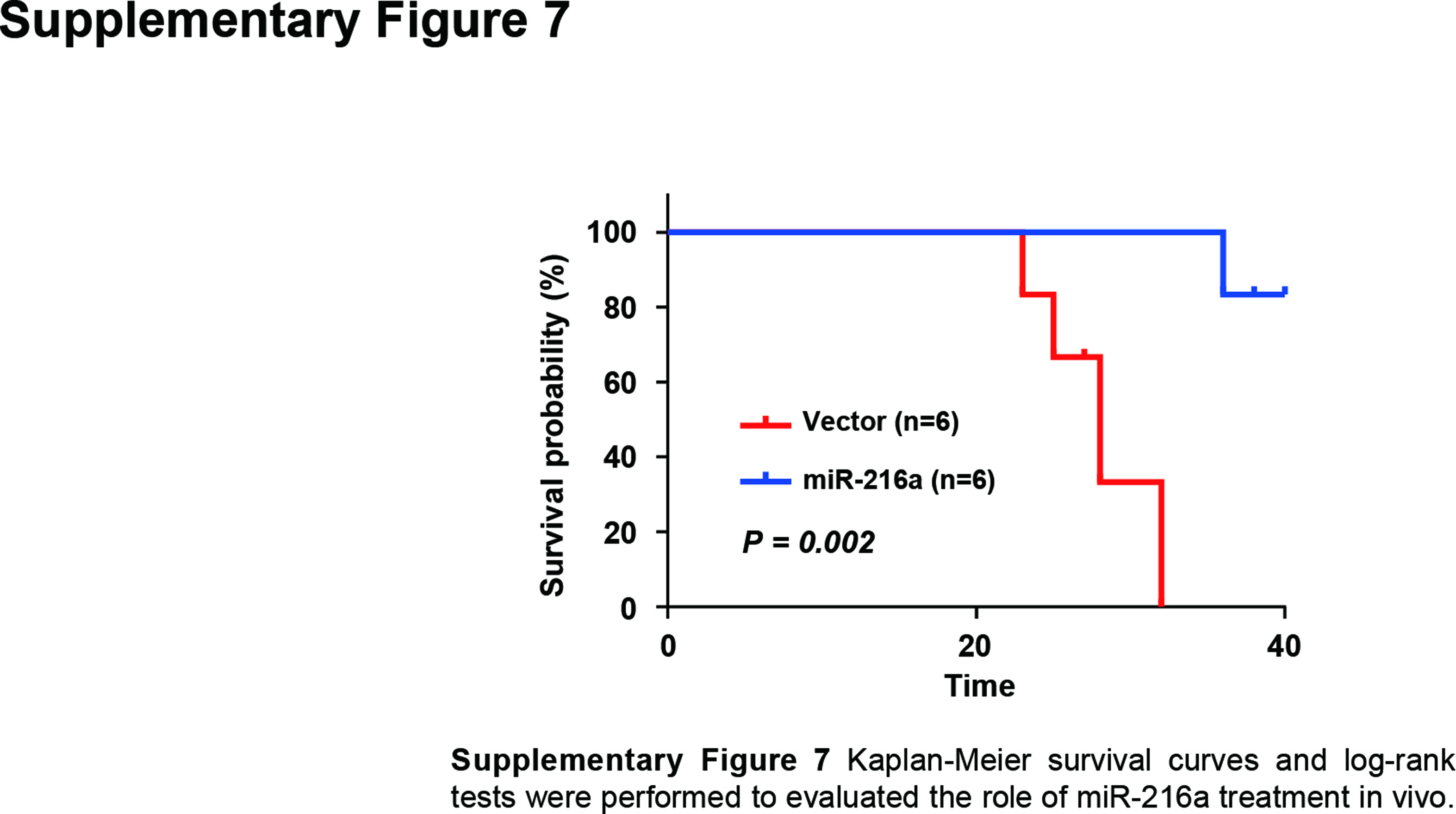

Supplement: Supplementary Figure 7 [file cddis2017499x10.tif]
